# DNA Barcoding Green Microalgae Isolated from Neotropical Inland Waters

**DOI:** 10.1371/journal.pone.0149284

**Published:** 2016-02-22

**Authors:** Sámed I. I. A. Hadi, Hugo Santana, Patrícia P. M. Brunale, Taísa G. Gomes, Márcia D. Oliveira, Alexandre Matthiensen, Marcos E. C. Oliveira, Flávia C. P. Silva, Bruno S. A. F. Brasil

**Affiliations:** 1 Embrapa Agroenergy, Brasília, DF, Brazil; 2 Universidade Federal do Tocantins, Gurupi, TO, Brazil; 3 Universidade Federal de Minas Gerais, Belo Horizonte, MG, Brazil; 4 Universidade Federal da Bahia, Vitória da Conquista, BA, Brazil; 5 Embrapa Pantanal, Corumbá, MS, Brazil; 6 Embrapa Swine and Poultry, Concórdia, SC, Brazil; 7 Embrapa Amazônia Oriental, Belém, PA, Brazil; ICGEB, INDIA

## Abstract

This study evaluated the feasibility of using the Ribulose Bisphosphate Carboxylase Large subunit gene (*rbc*L) and the Internal Transcribed Spacers 1 and 2 of the nuclear rDNA (*nu*ITS1 and *nu*ITS2) markers for identifying a very diverse, albeit poorly known group, of green microalgae from neotropical inland waters. Fifty-one freshwater green microalgae strains isolated from Brazil, the largest biodiversity reservoir in the neotropics, were submitted to DNA barcoding. Currently available universal primers for ITS1-5.8S-ITS2 region amplification were sufficient to successfully amplify and sequence 47 (92%) of the samples. On the other hand, new sets of primers had to be designed for *rbc*L, which allowed 96% of the samples to be sequenced. Thirty-five percent of the strains could be unambiguously identified to the species level based either on *nu*ITS1 or *nu*ITS2 sequences’ using barcode gap calculations. *nu*ITS2 Compensatory Base Change (CBC) and ITS1-5.8S-ITS2 region phylogenetic analysis, together with morphological inspection, confirmed the identification accuracy. In contrast, only 6% of the strains could be assigned to the correct species based solely on *rbc*L sequences. In conclusion, the data presented here indicates that either *nu*ITS1 or *nu*ITS2 are useful markers for DNA barcoding of freshwater green microalgae, with advantage for *nu*ITS2 due to the larger availability of analytical tools and reference barcodes deposited at databases for this marker.

## Introduction

DNA barcoding is a method used for species identification, which identifies specimens based on DNA sequence similarity against a sequence database of *a priori* defined species[1]. This powerful technique has brought significant improvements to applications such as taxonomy [2–4], ecology [5, 6], biosecurity [7–9] and food product regulation [10–12]. DNA-based identification is particularly useful for unveiling cryptic diversity at various taxonomic levels and identifying species where there are few or difficult to observe structural characters [13–17].

The green algae, Chlorophyta, are an ancient and taxonomically diverse lineage with approximately 8,000 described species [18, 19]. It is estimated that at least 5,000 species still remain undescribed, notably in tropical and subtropical areas [19]. Chlorophytes are important producers in aquatic and humid terrestrial ecosystems, which are often used as bioindicators in water monitoring and ecological studies [20, 21]. In addition, there is a growing interest in using green microalgae for biotechnological applications such as the production of fuels, chemicals, food and animal feed [22, 23]. The identification of green microalgae can be a difficult task and often requires careful microscopic examination of live cultured cells by a trained specialist [14, 24, 25]. Even so, the presence of cryptic species and phenotypic plasticity found in some species may hamper conclusive morphologic species diagnosis [26, 27]. DNA barcodes could provide the means to identify green microalgae consistently and rapidly, regardless of life stage [13, 28, 29].

Targets for potential Chlorophyta DNA barcodes have included chloroplast (*rbc*L, *tuf*A and Cp23S), mitochondrial (COI) and nuclear genes (18S rDNA, *nu*ITS1 and *nu*ITS2) [13, 28–30]. However, none of these markers were considered ideal for use across all lineages tested [13, 29, 31, 32]. Given the complexity and heterogeneity of chlorophytes, the protist working group of the Consortium for the Barcode of Life (CBOL) recommended the use of a two-step barcoding pipeline in which a universal pre-barcode marker should be used first, followed by the use of a group-specific second barcode [29]. A dual marker barcode based on *mat*K and *rbc*L genes has been formally proposed for use in DNA barcoding embryophytes [4]. However, the *mat*K gene is absent in chlorophytes precluding its use in this group [33]. Despite the unavailability of a universal PCR toolkit for *rbc*L amplification, this marker is considered a promising barcode for green algae [13]. Indeed, there are currently 4,449 *rbc*L sequences from chlorophyte species deposited at the Barcode of Life Data Systems (BOLD), a taxonomically curated database [3]. Apart from *rbc*L, the most promising candidates for green microalgae barcoding are the *nu*ITS1 and *nu*ITS2 markers [13, 14, 26, 28, 30, 34]. The ITS1-5.8S-ITS2 region from virtually all Viridiplantae can be amplified with a single set of universal primers [35], despite these being markers of high variability [13]. Furthermore, it is possible to analyze not only the *nu*ITS1 and *nu*ITS2 primary sequence, but also their secondary structures [36]. Although there are reports indicating that *nu*ITS1 and *nu*ITS2 might be insufficiently conserved or confounded by introgression or biparental inheritance patterns, a growing body of evidence has shown that simultaneous analysis of nucleotide data and compensatory base changes (CBCs) with secondary structure information can overcome most of the limitations of this potential barcode [14, 28, 30]. In addition, *nu*ITS1 and *nu*ITS2 have been the molecular markers of choice in several recent taxonomic revisions of freshwater chlorophytes species that were based on integrated morphological, physiological and molecular approaches [14, 26, 27, 34, 37–42] The use of *nu*ITS1- and *nu*ITS2-based phylogenies promoted considerable changes in green microalgae taxonomy, especially in taxa with simple morphology and few ultrastructural characteristics such as coccoid chlorophytes [26, 27].

This study aimed to identify neotropic green microalgae specimens isolated from Brazilian inland waters through the use of *rbc*L, *nu*ITS1 and *nu*ITS2 molecular markers as DNA barcodes. Brazilian continental waters comprise a biodiversity reservoir of enormous global significance and might contain up to 25% of the world’s algae species [43]. Novel primers for neotropic specimens’ *rbc*L gene amplification and sequencing are presented, as well as comparisons between *rbc*L, *nu*ITS1 and *nu*ITS2 markers variability, primers universality and databases accuracy and comprehensiveness.

## Materials and Methods

### Isolation and culturing

All the sample collections were made under the authorization SISBIO #39146 (09/26/2013) conceded by the Instituto Chico Mendes de Conservação da Biodiversidade (ICMBio) of the Brazillian Ministry of the Environment (MMA). The collections made on private land were also authorized by the owner of the land. This study did not involve endangered or protected species. Water samples were collected from the sites shown in S1 Fig. The collection environments included natural freshwater bodies within the Amazon rainforest, the Cerrado savanna and the Pantanal flooded grasslands, as well as anthropogenic wastewater deposits from the sugarcane industry (vinasse), pisciculture ponds and wastewater from swine farming. Sampling areas were delimited as being a 1 km radius centered in the geographic coordinates shown in S1 Fig. The collected environmental samples were submitted to an enrichment step through suspension in modified Bold's Basal Medium–BBM [44] and subsequent culturing at 28°C, light intensity of 50 μEm^-2^ s^-1^ and 16/8h light/dark regime. After 15 days of culture, the microalgae strains were isolated by two subsequent rounds of subculturing on BBM agar plates supplemented with ampicillin (100 μg/ml), chloramphenicol (25 μg/ml) and amphotericin B (2,5 μg/ml) under the same conditions described above. Individualized macroscopic colonies on agar plates were collected and inoculated into liquid BBM media to derive axenic cultures. The absence of contaminants was confirmed through microscopic inspection. The isolated strains were deposited in the Collection of Microorganisms and Microalgae Applied to Agroenergy and Biorefineries at Embrapa (Brasília/DF–Brazil).

### DNA extraction, amplification and sequencing

Total genomic DNA was isolated from 30 mg of fresh algal biomass using the Cetyl Trimethylammonium Bromide (CTAB) DNA extraction protocol adapted by [45]. The *rbc*L and ITS1-5.8S-ITS2 DNA regions were submitted to PCR amplification using the primers described in Table 1. The 25 μL PCR reaction mix was composed of 14.5 μL of ultrapure water, 5 μL of GoTaq 5X PCR buffer, 1.5 μL MgCl_2_ 25 mM, 0.75 μL BSA 10 mg/mL, 0.5 μL dNTPs 10 mM, 0.25 μL of GoTaq DNA polymerase (5 U/μL) (Promega, USA), 0.25 μL of each primer (10 μM) and 2.0 μl of DNA template (50–100 ng/μL). The PCR amplification protocol used for both markers was: 96°C for 5 min, 40 cycles of 96° C for 1 min, (primer annealing temperature—see Table 1) for 1 min and 72°C for 1 min, with a final extension at 72° C for 5 min. The PCR products (5 μL) were visualized on agarose gels and selected for direct sequencing. Sequences were determined bi-directionally for at least two different amplicons using the BigDye Terminator v.3.1 Cycle Sequencing Kit on the ABI 3130 automated DNA sequencer (both from Life Technologies, USA), in accordance with the manufacturer’s instructions. The forward and reverse sequences were aligned and edited using Geneious 6.1 software [46], generating consensus nucleotide positions with QV ≥ 20. Sequences were deposited in GenBank under the accession numbers: *rbc*L sequences (KT307991 to KT308039); ITS1-5.8S-ITS2 sequences (KT308040 to KT308042; KT308046 to KT308076; KT308078 to KT308086; KT445859 to KT445863).

**Table 1:** List of primers used in this study, including the primer sequences, amplicon length, annealing temperature and the sequencing success rate for a total of 51 strains tested.

Primer pair	Molecular marker	Sequence	Amplicon length (Nucleotides span)	Annealing temperature	Sequencing success rate	Reference
Fw_ITS1/Rv_ITS4	ITS1-5.8S-ITS2	Fw_ITS1: 5’–AGGAGAAGTCGTAACAAGGT– 3’ Rv_ITS4: 5’–TCCTCCGCTTATTGATATGC– 3’	≈ 650 pb	52°C	92,15%	[35]
Fw_rbcL_192/Rv_rbcL_657	rbcL	Fw_rbcL_192: 5’–GGTACTTGGACAACWGTWTGGAC– 3’ Rv_rbcL_657: 5’–GAAACGGTCTCKCCARCGCAT– 3’	465 pb (position 192 to 657)	52°C	82,35%	This study
Fw_rbcL_375/Rv_rbcL_1089	rbcL	Fw_rbcL_375: 5’–TTTGGTTTCAAAGCIYTWCGTGC– 3’ Rv_rbcL_1089: 5’–ATACCACGRCTACGRTCTTT– 3’	714 pb (position 375 to 1089)	52°C	50,98%	This study
Fw_rbcL_192/Rv_rbcL_1089	rbcL	Fw_rbcL_192: 5’–GGTACTTGGACAACWGTWTGGAC– 3’ Rv_rbcL_1089: 5’–ATACCACGRCTACGRTCTTT– 3’	897 pb (position 192 to 1089)	52°C	37,25%	This study
Fw_rbcLa_f/Rv_rbcL_ajf634R	rbcL	Fw_rbcLa_f 5’–ATGTCACCACAAACAGAAACTAAAGC– 3’ Rv_rbcL_ajf634R: 5’–GAAACGGTCTCTCCAACGCAT– 3’	654 pb (position 1 to 654)	54°C	15,69%	[4]
Fw_rbcL_109/Rv_rbcL_657	rbcL	Fw_rbcL_109: 5’–TTCTTGCTGCITTYCGTATG– 3’ Rv_rbcL_657: 5’–GAAACGGTCTCKCCARCGCAT– 3’	548 pb (position 109 to 657)	52°C	13,75%	This study
Fw_rbcLa_f/rbcLA_rev	rbcL	Fw_rbcLa_f: 5’–ATGTCACCACAAACAGAGACTAAAGC– 3’ rbcLA_rev: 5’–GTAAAATCAAGTCCACCRCG– 3’	599 pb (position 1 to 599)	54°C	7,84%	[4]
Fw_rbcL_109/Rv_rbcL_1089	rbcL	Fw_rbcL_109: 5’–TTCTTGCTGCITTYCGTATG– 3’ Rv_rbcL_1089: 5’–ATACCACGRCTACGRTCTTT– 3’	980 pb (position 109 to 1089)	52°C	1,96%	This study
Fw_rbcL_RH1/rbcL_724R	rbcL	Fw_rbcL_RH1: 5’–ATGTCACCACAAACAGAAACTAAAGC– 3’ rbcL_724R: 5’–TCGCATGTACCTGCAGTAGC– 3’	743 pb (position 1 to 743)	54°C	1,96%	[4]
Fw_rbcL_RH1/rbcL_1385R	rbcL	Fw_rbcL_RH1: 5’–ATGTCACCACAAACAGAAACTAAAGC– 3’ rbcL_1385R: 5’–AATTCAAATTTAATTTCTTTCC– 3’	1406 pb (position 1 to 1406)	48°C	0%	[13]

### Molecular data analysis

Sequences were aligned automatically using ClustalW [47] under default parameters using MEGA5 software [48]. The *nu*ITS1, 5.8S and *nu*ITS2 sequences were annotated using ITSx v. 1.0.11 [49]. For similarity searches, the *rbc*L sequences were submitted to the Barcode of Life Data Systems (BOLD systems) using the *Plant identification* tool, while *nu*ITS2 sequences were submitted to the Basic Local Alignment Search Tool (BLASTN) for comparisons against nucleotide sequences deposited at the Genbank. The *nu*ITS2 secondary structures were predicted by either direct fold (energy minimization) or homology modelling [50]. Subsequently, in order to locate hemi-compensatory base changes (hemi-CBCs) and compensatory base changes (CBCs), each sequence-structure along with its top match on *ITS2 Blast* tool were aligned and analyzed with 4SALE v. 1.7 [51, 52].

The barcode gap was inferred based on uncorrected pair–wise (*p*) distance matrices. MEGA5 software was used for calculation. The taxon samplings used were reference *nu*ITS1, *nu*ITS2 and *rbc*L sequences derived from recent taxonomic revisions of the *Chlorella* and *Desmodesmus* genera [14, 53, 54] (S1–S3 Tables). The maximum intraspecific distances and minimum interspecific distances obtained were computed.

For phylogenetic tree analysis, the ITS1-5.8S-ITS2 sequences from Embrapa|LBA#2–3, #22–23, #26–27, #30, #32–36, #39, #42–44 and #50 strains were included in the dataset together with their respectively closest sequences at GenBank. *Desmodesmus* sp., *Chlorella* sp. and *Micractinium* sp. ITS1-5.8S-ITS2 reference sequences [14, 39, 53–55]. The dendrograms were constructed through the maximum likelihood (ML) method using MEGA5 software. The GTR model with invariable sites (I) and gamma distribution shape parameter (G) was chosen. The neighbor-joining (NJ) algorithm was used to generate the initial tree for ML computation. A phylogenetic test using the Bootstrap method (1,000 replicates) was used.

### Morphologic Identification

Microscopic morphologic identification at the genus level was performed according to Bellinger & Sigee, 2015 [56]. Further identification to species levels was accomplished by comparison with the species original descriptions that are available at the AlgaeBase [57]. In the case of the as of yet undescribed species, the morphological comparisons were made with the closest strains obtained in the molecular identification step: *Desmodesmus* sp. MAT2008c [58]; *Micractinium* sp. CCAP 211/92 [39]; *Desmodesmus* sp. GM4a [59]. A Carl Zeiss Axio Imager A2 microscope (Zeiss.co, Brazil) equipped with Differential Interference Contrast (DIC) was used for morphological analysis.

## Results

### Barcode markers primer universality

A total of 51 unialgal strains (named Embrapa|LBA#1 to #51) were isolated from natural water bodies within the Cerrado savanna, the Pantanal wetlands and the Amazon rainforest, as well as anthropogenic wastewater deposits (S1 Fig). Coccoid morphotypes were the most abundant among the isolated strains (51%), followed by monadoids/palmelloids morphotypes (41%) (data not shown).

The ITS1-5.8S-ITS2 region could be successfully sequenced from DNA samples extracted from 47 strains (92,15% sequencing success rate) by using the universal primers described by White and coworkers (1990) [35] (Table 1). Even though all the 51 samples could be amplified with this set of primers, the presence of multiple PCR products impaired direct sequencing of four samples. On the other hand, the sequencing success rate obtained using the *rbc*L gene universal primer sets described by Hall and coworkers (2010) [13] or the sets proposed for embryophytes by the CBOL Plant working group [4], ranged from 0% to 15,69% (Table 1). In order to circumvent this problem, new sets of primers targeting *rbc*L gene partial amplification (Table 1) were designed based on 175 *rbc*L reference sequences from distinct Chlorophyta taxa mined from BOLD Systems. The newly designed primer pairs *Fw_rbcL_192/Rv_rbcL_657* and *Fw_rbcL_357/Rv_rbcL-1089* could successfully amplify and sequence 82,35% and 50,98% of the dataset, respectively (Table 1). The combination of the sequencing results from both these *rbc*L primer pairs allowed the construction of quality consensus sequences (QV≥20) for 49 samples (96,08% sequencing success rate). A total of 18 distinct 5.8S genotypes, 23 distinct *nu*ITS1 genotypes, 23 *nu*ITS2 distinct genotypes and 26 distinct *rbc*L genotypes were obtained.

### Similarity search based on *nu*ITS1, *nu*ITS2 and *rbc*L markers

In order to perform the molecular identification of Embrapa|LBA strains, the *rbc*L sequences obtained were submitted to similarity searches against the DNA barcoding dedicated database, BOLD systems. The closest matches retrieved for *rbc*L sequences ranged from 90% to 99% of similarity (Table 2). Currently, there are very few *nu*ITS1 and *nu*ITS2 sequences from chlorophytes deposited at taxonomically curated databases such as BOLD, therefore similarity searches were performed against the GenBank. The closest matches retrieved for *nu*ITS1 sequences ranged from 70% to 100% of similarity and for *nu*ITS2 sequences ranged from 81% to 100% of similarity (Table 2). Embrapa|LBA strains retrieved matches from species that belong to the Chlorophyceae and Trebouxiophyceae classes, especially to the orders Chlamydomonadales, Chlorococcales, Sphaeropleales and Chlorellales (Table 2). Ten *nu*ITS1 sequences, 14 *nu*ITS2 sequences and 0 *rbc*L sequences retrieved matches with a 100% similarity (Table 2).

**Table 2:** Molecular identification of the strains used in this study, including the percentual of identity, accession number and the name of the identified species on the Barcode of Life Database (based on *rbc*L marker sequence) and GenBank (based on *nu*ITS2 marker sequence).

Strain	ITS1 (GenBank)			ITS2 (GenBank)			*rbc*L (BOLD)			
	Closest match species	Identity	GenBank access	Closest match species	Identity	Number of CBCs / hCBCs	GenBank access	Closest match species	Identity	GenBank access
LBA#1	*Desmodesmus armatus*	95%	KP281288.1	*Desmodesmus bicellularis*	91%	1 / 7	AB917134.1	*Scenedesmus quadricauda*	90%	AB084332.1
LBA#2	*Desmodesmus* sp. MAT-2008c	100%	EU502836.1	*Desmodesmus* sp. MAT-2008c	100%	0 / 0	EU502836.1	*Acutodesmus obliquus*	93%	DQ396875.1
LBA#3	*Desmodesmus* sp. MAT-2008c	100%	EU502836.1	*Desmodesmus* sp. MAT-2008c	100%	0 / 0	EU502836.1	*Acutodesmus obliquus*	90%	DQ396875.1
LBA#4	*Chlamydopodium starrii*	70%	AB983644.1	*Chlorococcum oleofaciens*	91%	1 / 2	AB983633.1	*Chlorococcum ellipsoideum*	91%	EF113431.1
LBA#5	*Desmodesmus* sp. Tow 10/11 T-12W	79%	DQ417556.1	*Desmodesmus regularis*	84%	4 / 2	AM228924.1	*Desmodesmus santosii*	93%	GU192417.1
LBA#6	*Chlamydopodium starrii*	70%	AB983644.1	*Chlorococcum oleofaciens*	94%	-	AB983633.1	*Chlorococcum ellipsoideum*	91%	EF113431.1
LBA#7	*Desmodesmus* sp. Tow 10/11 T-12W	79%	DQ417556.1	*Desmodesmus regularis*	84%	4 / 2	AM228924.1	*Desmodesmus santosii*	93%	GU192417.1
LBA#8	*Chlamydomonas* sp. KU107	94%	KM061447.1	*Chlamydomonas* sp. KU107	87%	0 / 1	KM061447.1	*Chlamydomonas oblonga*	95%	EF113424.1
LBA#9	*Chlamydopodium starrii*	90%	AB983644.1	*Chlamydopodium starrii*	93%	0 / 1	AB983644.1	*Chlorococcum ellipsoideum*	92%	KC810301.1
LBA#10	*Chlamydopodium starrii*	90%	AB983644.1	*Chlamydopodium starrii*	93%	0 / 1	AB983644.1	*Chlorococcum ellipsoideum*	92%	KC810301.1
LBA#11	*Chlamydopodium starrii*	90%	AB983644.1	*Chlamydopodium starrii*	93%	0 / 1	AB983644.1	*Chlorococcum ellipsoideum*	92%	KC810301.1
LBA#12	*Chlamydopodium starrii*	90%	AB983644.1	*Chlamydopodium starrii*	93%	0 / 1	AB983644.1	*- *	-	-
LBA#13	*Coelastrella* sp. shy-188	96%	KP702302.1	*Scenedesmus rubescens*	95%	0 / 2	JX513884.1	*Scenedesmus quadricauda*	90%	AB084332.1
LBA#14	*Chlamydopodium starrii*	90%	AB983644.1	*Chlamydopodium starrii*	93%	0 / 1	AB983644.1	*Chlorococcum ellipsoideum*	92%	KC810301.1
LBA#15	*Chlamydopodium starrii*	90%	AB983644.1	*Chlamydopodium starrii*	93%	0 / 1	AB983644.1	*Chlorococcum ellipsoideum*	92%	KC810301.1
LBA#16	*-*	-	-	*-*	-	-	-	*Ecballocystopsis dichotomus*	90%	JX018187.1
LBA#17	*Chlamydopodium starrii*	90%	AB983644.1	*Chlamydopodium starrii*	93%	0 / 1	AB983644.1	*Chlorococcum ellipsoideum*	92%	KC810301.1
LBA#18	*Chlamydopodium starrii*	90%	AB983644.1	*Chlamydopodium starrii*	93%	0 / 1	AB983644.1	*Chlorococcum ellipsoideum*	92%	KC810301.1
LBA#19	*-*	-	-	*-*	-	-	-	*Ecballocystopsis dichotomus*	90%	JX018187.1
LBA#20	*Coelastrum astroideum*	76%	GQ375093.1	*Scenedesmus arcuatus*	81%	0 / 6	AY170855.1	*Hariotina reticulata*	93%	JQ394815.1
LBA#21	*Coelastrella* sp. shy-188	96%	KP702302.1	*Scenedesmus rubescens*	95%	0 / 2	JX513884.1	*Desmodesmus costato-granulatus*	94%	GU192427.1
LBA#22	*Desmodesmus ultrasquamatus*	100%	GU192392.1	*Desmodesmus ultrasquamatus*	99%	0 / 0	GU192392.1	*Desmodesmus costato-granulatus*	93%	GU192427.1
LBA#23	*Desmodesmus ultrasquamatus*	100%	GU192392.1	*Desmodesmus ultrasquamatus*	99%	0 / 0	GU192392.1	*Desmodesmus costato-granulatus*	94%	GU192427.1
LBA#24	*Desmodesmus ultrasquamatus*	94%	GU192392.1	*Desmodesmus ultrasquamatus*	94%	0 / 3	AM228926.1	*Desmodesmus costato-granulatus*	94%	GU192427.1
LBA#25	*Desmodesmus ultrasquamatus*	94%	GU192392.1	*Desmodesmus ultrasquamatus*	94%	0 / 3	AM228926.1	*Desmodesmus costato-granulatus*	94%	GU192427.1
LBA#26	*Desmodesmus* sp. MAT-2008c	100%	EU502836.1	*Desmodesmus* sp. MAT-2008c	100%	0 / 0	EU502836.1	*Acutodesmus obliquus*	92%	DQ396875.1
LBA#27	*Chlorella sorokiniana*	100%	KM061456.1	*Chlorella sorokiniana*	100%	0 / 0	KJ676113.1	*Chlorella sorokiniana*	99%	HM101339.1
LBA#28	*-*	-	-	*-*	-	-	-	*Selenastrum* sp. KMMCC 1456	94%	JQ315488.1
LBA#29	*Chlorella* sp. MAT-2008a	92%	EU502833.1	*Chlorella* sp. MAT-2008a	91%	0 / 2	EU502833.1	*Chlorella* sp. IFRPD 1018	93%	AB260911.1
LBA#30	*Desmodesmus* sp. MAT-2008c	100%	EU502836.1	*Desmodesmus* sp. MAT-2008c	100%	0 / 0	EU502836.1	*Acutodesmus obliquus*	93%	DQ396875.1
LBA#31	*Chlorella* sp. MAT-2008a	92%	EU502833.1	*Chlorella* sp. MAT-2008ª	91%	0 / 2	EU502833.1	*Chlorella* sp. IFRPD 1018	93%	AB260911.1
LBA#32	*Micractinium* sp. CCAP 211/92	99%	FM205863.1	*Micractinium* sp. CCAP 211/92	100%	0 / 0	FM205863.1	*Chlorella pyrenoidosa*	99%	FM205863.1
LBA#33	*Micractinium* sp. CCAP 211/92	99%	FM205863.1	*Micractinium* sp. CCAP 211/92	100%	0 / 0	FM205863.1	*Chlorella pyrenoidosa*	99%	FM205863.1
LBA#34	*Micractinium* sp. CCAP 211/92	99%	FM205863.1	*Micractinium* sp. CCAP 211/92	100%	0 / 0	FM205863.1	*Chlorella pyrenoidosa*	99%	FM205863.1
LBA#35	*Desmodesmus* sp. GM4a	100%	AB917128.1	*Desmodesmus* sp. GM4a	99%	0 / 1	AB917128.1	*Desmodesmus baconii*	93%	KC315289.1
LBA#36	*Desmodesmus* sp. MAT-2008c	100%	EU502836.1	*Desmodesmus* sp. MAT-2008c	100%	0 / 0	EU502836.1	*Acutodesmus obliquus*	93%	DQ396875.1
LBA#37	*Chlamydomonas* sp. YB3-2	90%	JN862852.1	*Chlamydomonas applanata*	92%	1 / 2	FR865616.1	*Ascochloris multinucleata*	94%	EF113411.1
LBA#38	*Chlamydomonas* sp. YB3-2	90%	JN862852.1	*Chlamydomonas applanata*	92%	1 / 2	FR865616.1	*Ascochloris multinucleata*	94%	EF113411.1
LBA#39	*Chlorella sorokiniana* KU207	100%	KM061456.1	*Chlorella sorokiniana*	100%	0 / 0	KJ676113.1	*Chlorella sorokiniana*	99%	HM101339.1
LBA#40	*Chlamydomonas zebra*	79%	AF033294.1	*Chlamydomonas* sp. XJU-36	95%	2 / 0	FJ572059.1	*Chlamydomonas orbicularis*	96%	AB511849.1
LBA#41	*Chlamydomonas* sp. KU107	94%	KM061447.1	*Chlamydomonas* sp. KU107	87%	0 / 3	KM061447.1	*Chlamydomonas oblonga*	95%	EF113424.1
LBA#42	*Micractinium* sp. CCAP 211/92	99%	FM205863.1	*Micractinium* sp. CCAP 211/92	100%	0 / 0	FM205863.1	*Chlorella pyrenoidosa*	99%	FM205863.1
LBA#43	*Micractinium* sp. CCAP 211/92	99%	FM205863.1	*Micractinium* sp. CCAP 211/92	100%	0 / 0	FM205863.1	*Chlorella pyrenoidosa*	99%	FM205863.1
LBA#44	*Micractinium* sp. CCAP 211/92	99%	FM205863.1	*Micractinium* sp. CCAP 211/92	100%	0 / 0	FM205863.1	*Chlorella pyrenoidosa*	99%	FM205863.1
LBA#45	*Chlorococcum oleofaciens*	82%	AB983633.1	*Spongiochloris spongiosa*	86%	-	U34776.1	*Protosiphon botryoides*	92%	EF113465.1
LBA#46	*Uronema* sp. AF-2012	98%	JX092263.1	*Uronema trentonense*	100%	0 / 0	HF920659.1	*-*	-	-
LBA#47	*Tetracystis tetraspora*	95%	KM020024.1	*Dunaliella* sp. SPMO 300–4	85%	2 / 0	DQ377118.1	*Nautococcus solutus*	91%	AB360758.1
LBA#48	*-*	-	-	*-*	-	-	-	*Gungnir* sp. NIES-1851	93%	AB603749.1
LBA#49	*Lobochlamys segnis*	83%	FR865604.1	*Chlamydomonas* sp. CCAP 11/150	90%	0 / 1	FR865545.1	*Asterococcus korschikoffii*	90%	AB175944.1
LBA#50	*Chlorella* sp. KMMCC 1468	99%	JQ315774.1	*Chlorella sorokiniana*	96%	0 / 0	LK021940.1	*Chlorella* sp. IFRPD 1014	99%	AB260910.1
LBA#51	*Chlorococcum oleofaciens*	74%	AB983630.1	*Chlorococcum* sp. CCAP 11/52	84%	2 / 1	FR865591.1	*Chlamydopodium vacuolatum*	95%	EF113426.1

The compensatory and hemi-compensatory base changes (CBCs/hemi-CBCs) between the indicated sequence and its closest match in the ITS2 Database are shown. An hyphen (-) is indicated for samples that could not be amplified and/or sequenced, and for the *nu*ITS2 sequences for which secondary structure predictions and CBCs/Hemi-CBCs analysis were not possible.

### Barcode gap analysis

Similarity searches only configure the first step for DNA barcoding since they provide information about the closest matches present in reference databases, but not necessarily species-level identification. In order to establish a genetic distance threshold for species-level identification that is applicable to chlorophytes, barcode gap analyses were conducted based on reference sequences from two species-dense green microalgae genera, *Chlorella* and *Desmodesmus* (S2–S4 Figs; S1–S3 Tables).

*Chlorella* genus *nu*ITS1 intraspecific distances ranged from 0 to 0,014, while *nu*ITS1 interspecific distances ranged from 0,058 to 0,199 (S2A Fig). *Desmodesmus* genus *nu*ITS1 intraspecific distances ranged from 0 to 0,018, while *nu*ITS1 interspecific distances ranged from 0,029 to 0,193 (S2B Fig). The presence of a barcode gap (gap between maximum intraspecific and minimum interspecific distances) was observed for all species analyzed (S2 Fig). *Chlorella* genus *nu*ITS2 intraspecific distances ranged from 0 to 0,071, while *nu*ITS2 interspecific distances ranged from 0,076 to 0,204 (S3A Fig). *Desmodesmus* genus *nu*ITS2 intraspecific distances ranged from 0 to 0,02, while *nu*ITS2 interspecific distances ranged from 0,032 to 0,167 (S3B Fig). The presence of a barcode gap was also observed for all species analyzed (S3 Fig). *Desmodesmus rbc*L genus intraspecific distances ranged from 0 to 0,108, while *rbc*L interspecific distances ranged from 0,015 to 0,086 (S4 Fig). The presence of a barcode gap is observed for all species based on *rbc*L sequences, except for *Desmodesmus serratus* species (S4 Fig).

Distance thresholds for species-level identification were inferred for each marker based on the minimum interspecific distances observed for each marker (S2–S4 Figs), as follows: i) *nu*ITS1 sequences (< 0,029); ii) *nu*ITS2 sequences (< 0,032); ii) *rbc*L sequences (< 0,015). The application of these distance thresholds to the data presented in Table 2 suggests that species-level identification has been achieved for: i) 35% of the *nu*ITS1 sequences, namely Embrapa|LBA#2–3, #22–23, #26–27, #30, #32–36, #39, #42–44, #46 and #50; ii) 33% of the *nu*ITS2 sequences, namely Embrapa|LBA#2–3, #22–23, #26–27, #30, #32–36, #39, #42–44 and #46. iii) 18% of the *rbc*L sequences, namely Embrapa|LBA#27, #32–34, #39, #42–44 and #50.

Additionally, even though *nu*ITS2 Embrapa|LBA#50 sequence presents only 96% of identity to its GenBank closest match, it can also be considered that species-level identification has been achieved, since the lowest interspecific distance calculated specifically for the *Chlorella* genus *nu*ITS2 sequences is 0,076 (S3A Fig). On the other hand, *rbc*L based identification assigned Embrapa|LBA #32–34 and #42–44 strains to *Chlorella pyrenoidosa* species, which is not currently a taxonomically accepted name [57]. Therefore, Embrapa|LBA #32–34 and #42–44 strains were excluded from the subset of strains identified to the species-level based on *rbc*L sequences.

In conclusion, the results presented so far indicate that 18, 18 and 3 Embrapa|LBA strains were identified to the species-level based on *nu*ITS1, *nu*ITS2 or *rbc*L sequences, respectively.

### Morphologic, Phylogenetic and Compensatory Base Changes (CBCs) analyses

**Fig 1:**
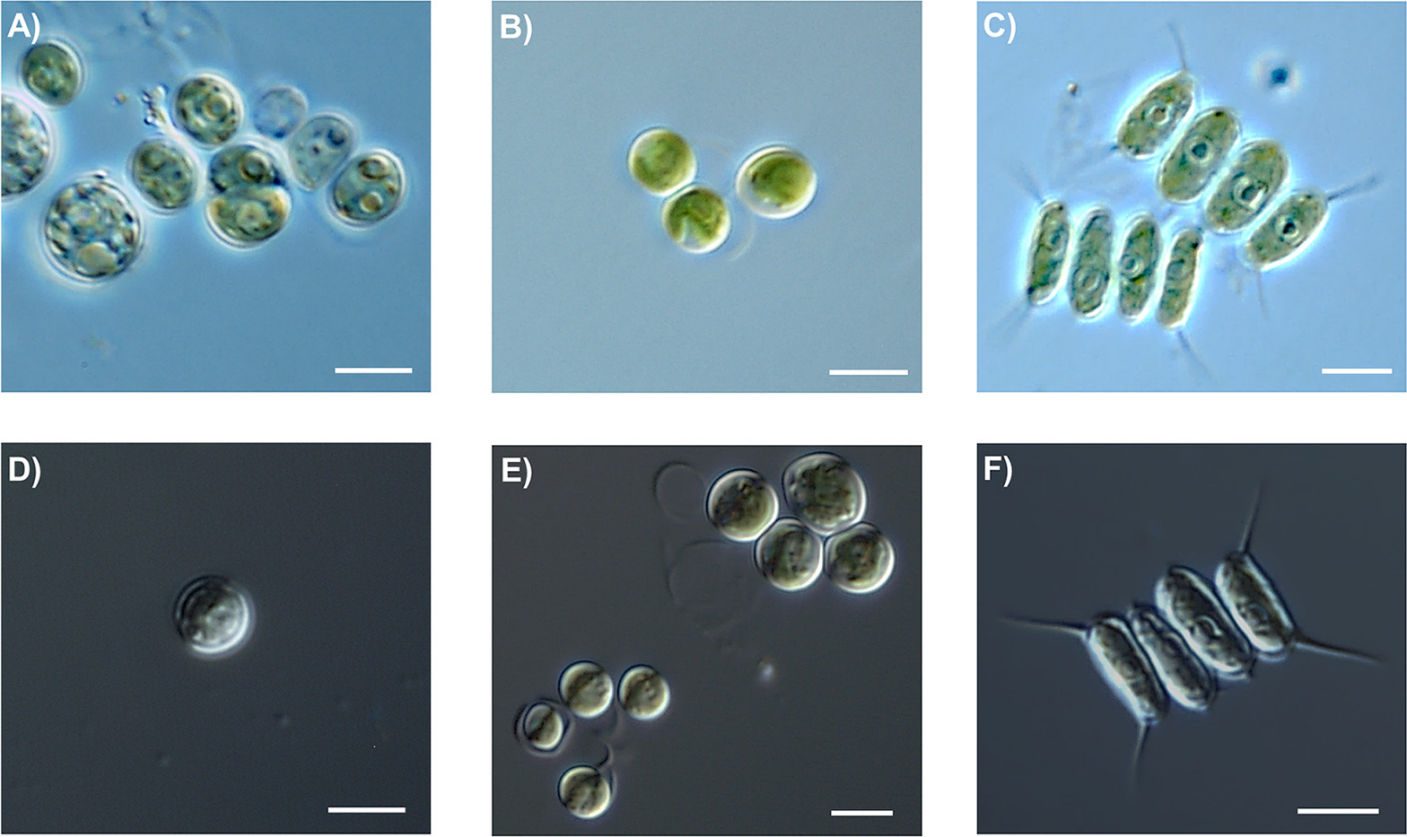
Representative DIC microscopic images of Embrapa|LBA strains assigned to not formally described species. (A and D) Embrapa|LBA#36. (B and E) Embrapa|LBA#32. (C and F) Embrapa|LBA#35. Scale bars = 5 μm.

In order to confirm the species-level identification based on barcode gap calculations, the strains Embrapa|LBA#2–3, #22–23, #26–27, #30, #32–36, #39, #42–44,#46 and #50 were identified based on morphology. The strains Embrapa|LBA#22–23 were identified as *Desmodesmus ultrasquamatus*, Embrapa|LBA#27, 39 and 50 were identified as *Chlorella sorokiniana* and Embrapa|LBA#46 was identified as *Uronema trentonense*, according to these species original descriptions [57]. The molecular identification of strains Embrapa|LBA#2–3, #26, #30, #32–36 and #42–44 (Table 2) suggest that they correspond to species still not formally described. Indeed, strains Embrapa|LBA#2–3, #26, #30 and #36 correspond to unicellular spineless coccoid *Desmodemus* species with sizes ranging from 4–6 μm (Fig 1A and 1D), similar to the description of its closest GenBank match (Table 2) the strain *Desmodesmus* sp. MAT-2008c isolated in Australia [58]. Strains Embrapa|LBA#32–34 and #42–44 correspond to coccoid bristleless *Micractinium* species with sizes ranging from 3–5 μm (Fig 1B and 1E), which is congruent with the description reported for its closest GenBank match (Table 2) the strain *Micractinium* sp. CCAP 211/92 isolated from a soil sample collected from Mahe Island, Seychelles [39]. Strain Embrapa|LBA#35 corresponds to a two-, four- or eight-celled coenobia forming *Desmodemus* species that present few spines and dimensions of 3–6 x 8–13 μm (Fig 1C and 1F), similar to the description of its closest GenBank match (Table 2) the strain *Desmodesmus* sp. GM4a isolated from German inland waters [59].

**Fig 2:**
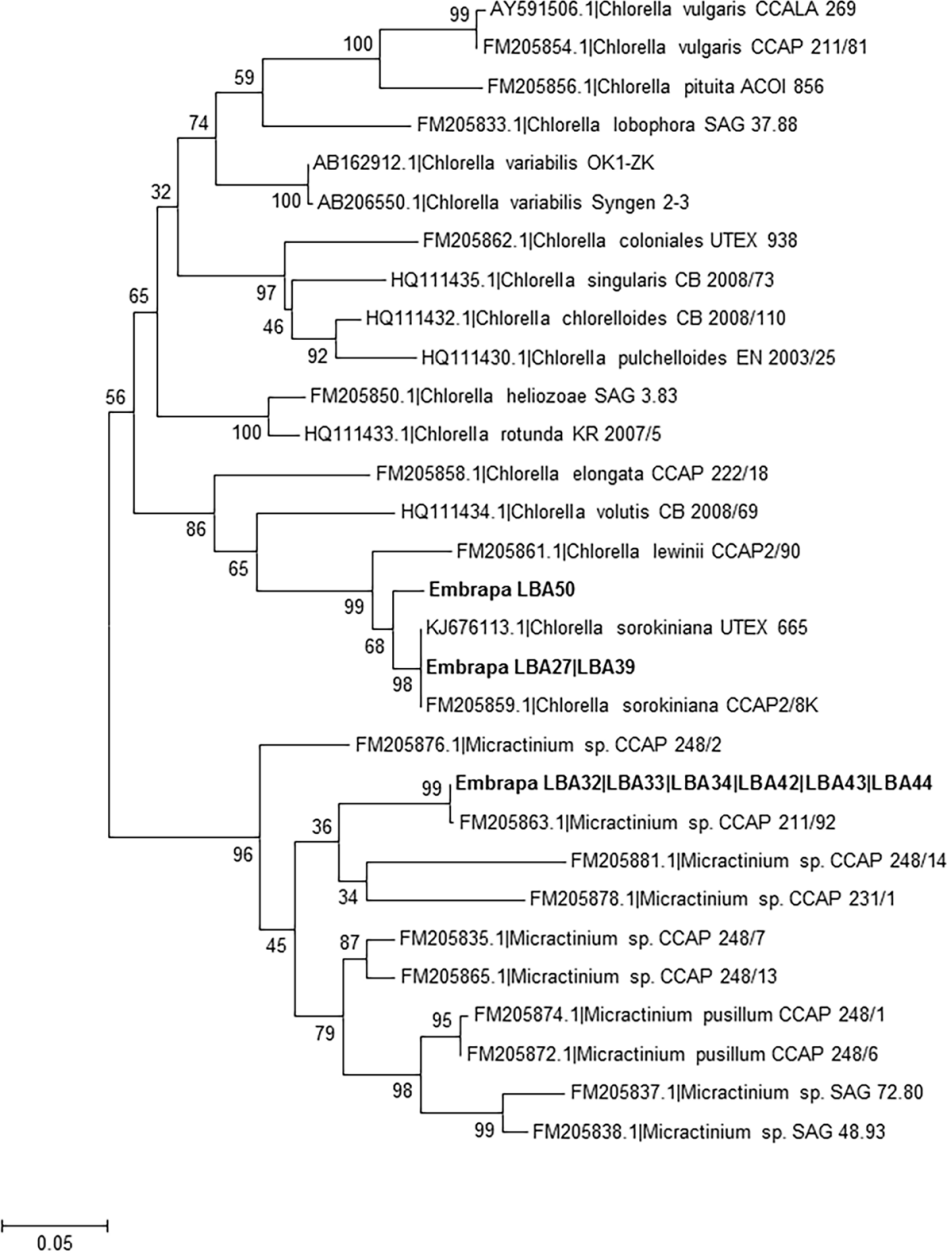
Phylogenetic tree for *Chlorella* and *Micractinium* genera inferred based on ITS1-5.8S-ITS2 sequences. *Chlorella* sp. and *Micractinium* sp. ITS1-5.8S-ITS2 reference barcode sequences reported by Luo et al. (2010) [39] and Bock et al. (2011) [14] were included in the analysis together with Embrapa|LBA#27, #32–34, #39, #42–44 and #50 strains sequences and their respectively closest sequences at GenBank. Identical sequences were omitted for simplification. The phylogenetic tree was inferred using the Maximum Composite Likelihood method based on dataset of 472 aligned positions of 31 nucleotide sequences. For the analysis, the GTR+G+I model was chosen. For the analysis, the GTR model with invariable sites (I) and gamma distribution shape parameter (G) was chosen. The bootstrap values (1000 replicates) are shown next to the branches.

**Fig 3:**
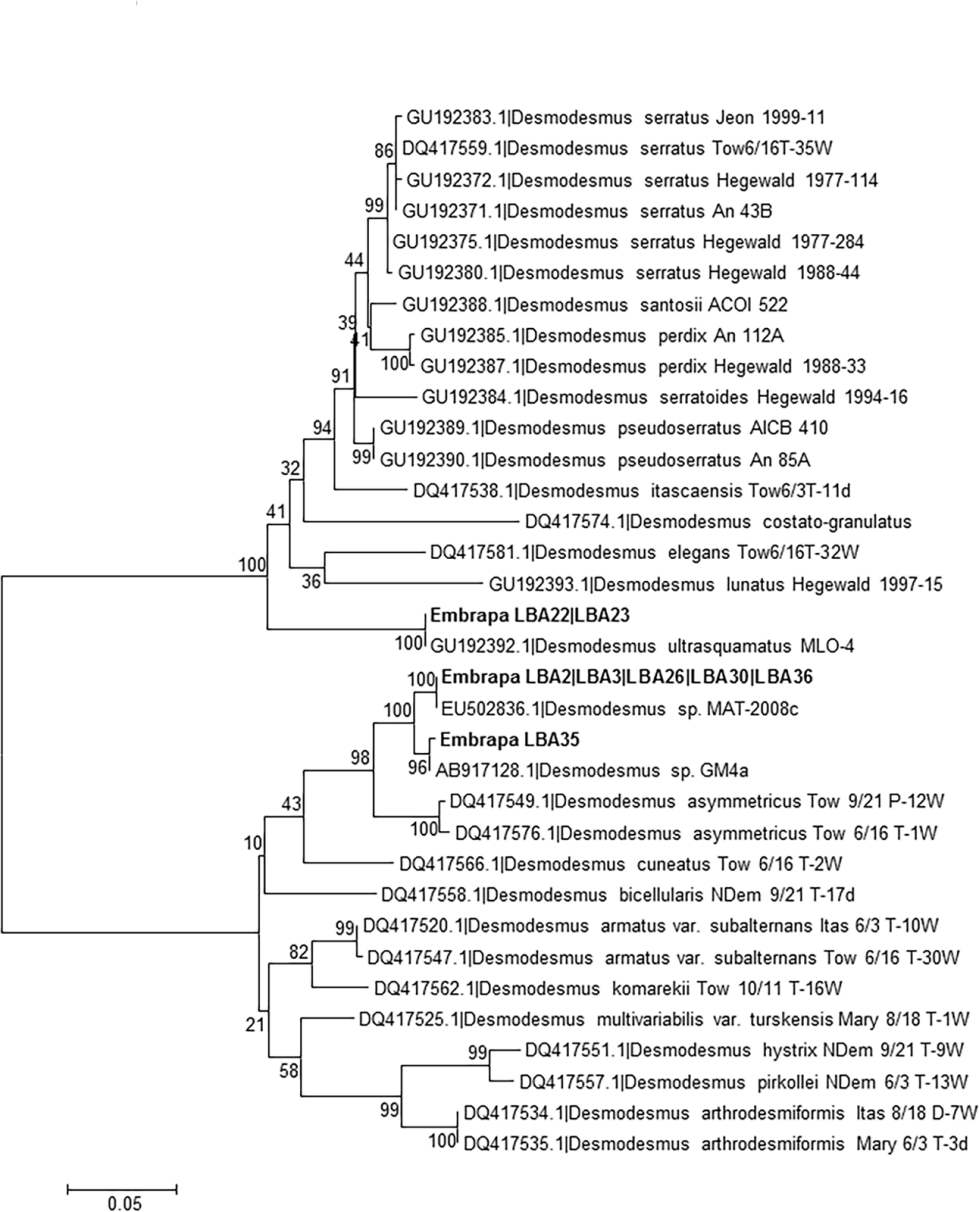
Phylogenetic tree for *Desmodesmus* genus inferred based on ITS1-5.8S-ITS2 sequences. *Demodesmus* sp. ITS1-5.8S-ITS2 reference barcode sequences reported by Fawley et al. (2011) [53] and Gorelova et al. (2014) [54] were included in the analysis together with Embrapa|LBA#2–3, #22–23, #26, #30 and #35–36 strains sequences and their respectively closest sequences at GenBank. Identical sequences were omitted for simplification. The phylogenetic tree was inferred using the Maximum Composite Likelihood method based on a dataset of 470 aligned positions of 34 nucleotide sequences. For the analysis, the GTR+G+I model was chosen. The bootstrap values (1000 replicates) are shown next to the branches.

Furthermore, the species-level identification obtained for strains Embrapa|LBA#2–3, #22–23, #26–27, #30, #32–36, #39, #42–44,#46 and #50 is corroborated by the absence of Compensatory Base Changes (CBCs) between *nu*ITS2 sequences of these strains and their closest matches at GenBank (Table 2). Additionally, phylogenetic analyses using reference ITS1-5.8S-ITS2 sequences from currently accepted *Chlorella*, *Micractinium* and *Desmodesmus* species also corroborate species-level identification of strains Embrapa|LBA#2–3, #22–23, #26–27, #30, #32–36, #39, #42–44 and #50 (Figs 2 and 3). Figs 2 and 3 clearly demonstrate that sequences from these strains group together with their closest matches from GenBank (Table 2) in monophyletic clades.

## Discussion

A dual marker DNA barcode system has been proposed as a potential solution to cope with the great diversity of protists, however there is no current consensus about which marker should be used [29, 32]. Ideally the two chosen markers should be easily amplified/sequenced using a single set of primers and sufficiently variable to permit clear species delimitations without loss of the phylogenetic signal [29, 32]. Even though *tuf*A has been reported to be a promising barcode for chlorophytes [13, 31, 32], the number of green algae *tuf*A sequences deposited at GenBank is three times lower than the number of deposits for the protein-coding plastid gene *rbc*L or the non-coding regions of nuclear rDNA ITS1 and ITS2 (over 6,000 sequences deposited for *rbc*L and *nu*ITS1 and over 7,000 sequences deposited for *nu*ITS2 markers up to December/2015). Furthermore, recent taxonomic revisions of green algae have been based mainly on *rbc*L, *nu*ITS1 or *nu*ITS2 sequences [14, 26, 27, 32, 34, 37–42, 60]. In addition, there are thousands of *rbc*L sequences from chlorophytes deposited at BOLD systems, which is the most complete taxonomically curated DNA database available [3]. Therefore, although a formal proposal for Chlorophyta DNA barcodes has not been made, a preference for *rbc*L, *nu*ITS1 and *nu*ITS2 markers by several research groups involved in green algae taxonomy can be observed.

Brazil holds the largest reservoir of algal genetic resources in the neotropical region [43, 61]. In order to evaluate the applicability of *nu*ITS1, *nu*ITS2 and *rbc*L markers as DNA barcodes for neotropic freshwater chlorophytes, a subset of green microalgae strains was isolated from Brazilian inland water bodies (S1 Fig). This study, however, did not intend to perform an exhaustive sampling of all the Chlorophyta taxa present in the neotropics. Instead, it used specimens from this largely unexplored biodiversity hotspot as test case. DNA from all 51 Embrapa|LBA strains could be amplified and sequenced for at least one of the markers tested. The higher primer universality obtained for ITS1-5.8S-ITS2 region compared to the *rbc*L marker (Table 1) is in agreement with previous studies [13, 28, 62]. This can be explained by the presence of highly conserved neighbor regions flanking *nu*ITS (1 and 2) markers, such as the 18S and 28S rDNA genes that function as annealing sites for the primers, described by White and coworkers (1990) [35], which are not available for the *rbc*L gene.

The levels of nucleotide diversity observed among the 5.8S, *nu*ITS1, *nu*ITS2 and *rbc*L sequences were of 0,046, 0,537, 0,321 and 0,250, respectively. Indeed, although *nu*ITS1, *nu*ITS2 and *rbc*L markers may fluctuate depending on the taxa analyzed, these markers rank among the most diverse barcode candidates for chlorophytes [13, 28, 31]. On the other hand, the 5.8S marker might not present sufficient resolution for species discrimination. Therefore, although other studies used the nuclear rDNA region ITS1-5.8S-ITS2 as a barcode for Chlorophyta (14, 34, 39), in this study the *nu*ITS1 and *nu*ITS2 regions were used separately to avoid genetic distance calculation bias eventually introduced by the simultaneous analysis of DNA regions with distinct evolutionary rates.

It is noteworthy that 53% of the *nu*ITS1 and 42% of the *nu*ITS2 matches retrieved from GenBank lacked the Latin binomial that characterizes the complete species name, compared to 10% of the *rbc*L matches retrieved from BOLD (Table 2). This might be due to the combination of two factors: i) CBOL’s effort to preserve traditional taxonomic nomenclature; ii) The overall tendency in phycology to gradually move away from species identifiers based on Latin binomials pushed by the faster rate of genetic information discovery compared with the traditional taxonomic descriptions [24]. Importantly, species names that are not currently taxonomically accepted were found at both the BOLD and GenBank databases. That is the case, for example, of the strains Embrapa|LBA#32–34 and #42–44, which were assigned as *Chlorella pyrenoidosa* (Table 2), currently *Pseudochlorella pyrenoidosa* [26, 38], at BOLD systems. Although this finding is not unexpected within GenBank, it is especially relevant in a taxonomically curated database such as BOLD. A possible explanation is that these are, actually, non-validated reference sequences mined directly from GenBank that are currently under taxonomic revision by BOLD collaborators. Indeed, it can be observed that the *Acutodesmus obliquus rbc*L reference sequence DQ396875.1 retrieved from BOLD (Table 2) is deposited with the old species name, *Scenedesmus obliquus*, at GenBank (data not shown).

Only few sequences retrieved matches with 100% of identity from GenBank and BOLD (Table2), suggesting incomplete taxa coverage within the reference databases analyzed. This is corroborated by the fact that there are less than 500 hundred *rbc*L records from the neotropical region (only 21 from Brazil) deposited at BOLD up to July/2015. Thus, it seems that the incongruences observed between species names retrieved from *nu*ITS1, *nu*ITS2 and *rbc*L similarity searches (Table 2) are mainly due to reference databases incompleteness rather than to real conflicts derived from distinct species identification by each marker. This is important information to be considered since the possibility of biased performance, eventually leading to sample misidentification, when using search algorithms such as BLAST is increased when analyzing poorly sampled groups [63].

Barcode gap analyses can provide the means to improve the accuracy for species level identification [1, 17]. A barcode gap is present when the maximum intraspecific distance is lower than the minimum interspecific distance for a certain taxon, thereby revealing a corresponding distance threshold that can be applied to delimit species [17]. However, the same distance threshold may not be applicable to every species and should be determined for each taxon analyzed [32, 63, 64]. Due to the unavailability of a complete set of reference sequences for most of the taxa listed in Table 2, the analyses were based on sequences *Chlorella* and *Desmodesmus* genera for *nu*ITS1 and *nu*ITS2, and for *Desmodesmus* genus for *rbc*L. These reliable reference barcode sequences are originated from recent revisions of these genera based on integrative taxonomy approaches (S2–S4 Figs; S1–S3 Tables). As expect, the barcode gap analyses based on *nu*ITS1, *nu*ITS2 and *rbc*L makers (S2–S4 Figs) indicate that it is not possible to establish a single universal distance threshold that would avoid incorrect identifications and, at the same time, include all specimens into the correct species. However, assuming that incorrect specimen identification is more problematic than simply not assigning a specimen to any species, distance thresholds were inferred for each marker based on the minimum interspecific distances observed (S2–S4 Figs) allowing species-level identification.

There are several reports suggesting that the presence of compensatory base changes (CBCs) in *nu*ITS2 secondary structures correlate with reproductive isolation [65–67]. A large-scale testing with ~300.000 *nu*ITS2 secondary structures revealed that if a CBC is present then there are two different species with a probability of ~93% [65, 67]. Therefore, the detection of CBCs between the Embrapa|LBA strains *nu*ITS2 sequences and their closest matches at GenBank seems to be a reasonable predictor that species-level identification has not been achieved. In accordance, the CBCs analyses shown in Table 2 corroborate the species-level identification achieved based on barcode gap calculations. Additionally, the morphological (Fig 1) and phylogenetic analyses (Figs 2 and 3) also corroborate the species-level identification based on barcode gap calculations.

The DNA barcoding results presented here using a subset of neotropic freshwater green microalgae as a test case suggest that *nu*ITS1 and *nu*ITS2 are the most useful markers, while *rbc*L presented lower primer universality and species-level identification power. Although, both *nu*ITS1 and *nu*ITS2 precisely identified the same 18 strains to the species-level based on barcode gap calculations, *nu*ITS2 accounts with a more complete set of reference sequences deposited at databases and an automated and well developed pipeline for secondary structure analysis [50]. The S5 Fig depicts the tentative DNA barcoding workflow for green microalgae specimens based on the results presented.

## Conclusions

DNA barcoding can make specimens identification to species level faster, more reliable and accessible to non-specialists. Defining of the appropriate DNA barcodes for Chlorophyta identification and the availability of taxonomically curated DNA databases are pivotal to this task. The results presented here indicate that a DNA barcoding pipeline based on *nu*ITS2 should be useful for green microalgae species identification. It is clear, however, that there is an urgent need for the deposition of more taxonomically accurate reference barcodes in curated databases (e.g.: BOLD Systems). Therefore, extensive efforts on integrative taxonomy are crucial, ideally encompassing the use of both DNA markers. These studies are especially relevant for poorly studied taxa such as tropical chlorophytes.

## Supporting Information

S1 Fig Collection sites. Map of Brazilian biomes, including the *Amazon* tropical rainforest (1), the *Caatinga* xeric shrublands (2), the *Cerrado* tropical Savanna (3), the *Pantanal* flooded grassland (4), the *Mata Atlântica* tropical rainforest (5) and the *Pampa* subtropical grassland (6). The geographic coordinates of the six distinct locations sampled and the respective isolated strains in each site are shown. The strains isolated were deposited in the Collection of Microorganisms and Microalgae Applied to Agroenergy and Biorefineries at Embrapa (Brasília/DF–Brazil). The Brazilian territory is highlighted in black in the map of the neotropical region (inset).
(TIF)

S2 Fig *nu*ITS1-based barcode gap calculation. The maximum intraspecific distances (◆) and minimum interspecific distances (□) based on *nu*ITS1 marker between *Chlorella* (A) and *Desmodesmus* (B) genera species are shown. The dataset was composed of reference barcode sequences reported for each genera (S1 and S2 Tables).
(TIF)

S3 Fig *nu*ITS2-based barcode gap calculation. The maximum intraspecific distances (◆) and minimum interspecific distances (□) based on *nu*ITS2 marker between *Chlorella* (A) and *Desmodesmus* (B) genera species are shown. The dataset was composed of reference barcode sequences reported for each genera (S1 and S2 Tables).
(TIF)

S4 Fig *rbc*L-based barcode gap calculation. The maximum intraspecific distances (◆) and minimum interspecific distances (□) based on *rbc*L marker between *Desmodesmus* genus species are shown. The dataset was composed of reference barcode sequences reported this genus (S3 Table).
(TIF)

S5 Fig Roadmap for green microalgae DNA barcoding. *nu*ITS2 should be primarily sequenced and submitted to similarity searches against GenBank. Similarity values obtained must be compatible with the barcode gap thresholds calculated using reference sequences for the taxon indicated (a). The absence of CBCs between the query *nu*ITS2 sequence and its closest match retrieved from similarity search is necessary to confirm species diagnosis (b). Finally, the current status of the assigned species name must be checked using a reference database (e.g.: AlgaeBase) (c). If *nu*ITS2 is not sufficient for a species diagnosis, other markers/methods should be tried (d).
(TIF)

S1 Table *nu*ITS1 and *nu*ITS2 reference sequences from *Chlorella* genus mined from GenBank used for barcode gap calculation. (DOCX)

S2 Table *nu*ITS1 and *nu*ITS2 reference sequences from *Desmodesmus* genus mined from GenBank used for barcode gap calculation. (DOCX)

S3 Table *rbc*L reference sequences from *Desmodesmus* genus mined from GenBank used for barcode gap calculation. (DOCX)
