# Supplementary figures and images for: DNA Barcoding Green Microalgae Isolated from Neotropical Inland Waters
Source: PLoS One. 2016 Feb 22;11(2):e0149284. doi: 10.1371/journal.pone.0149284 (PMC4767179; doi:10.1371/journal.pone.0149284)

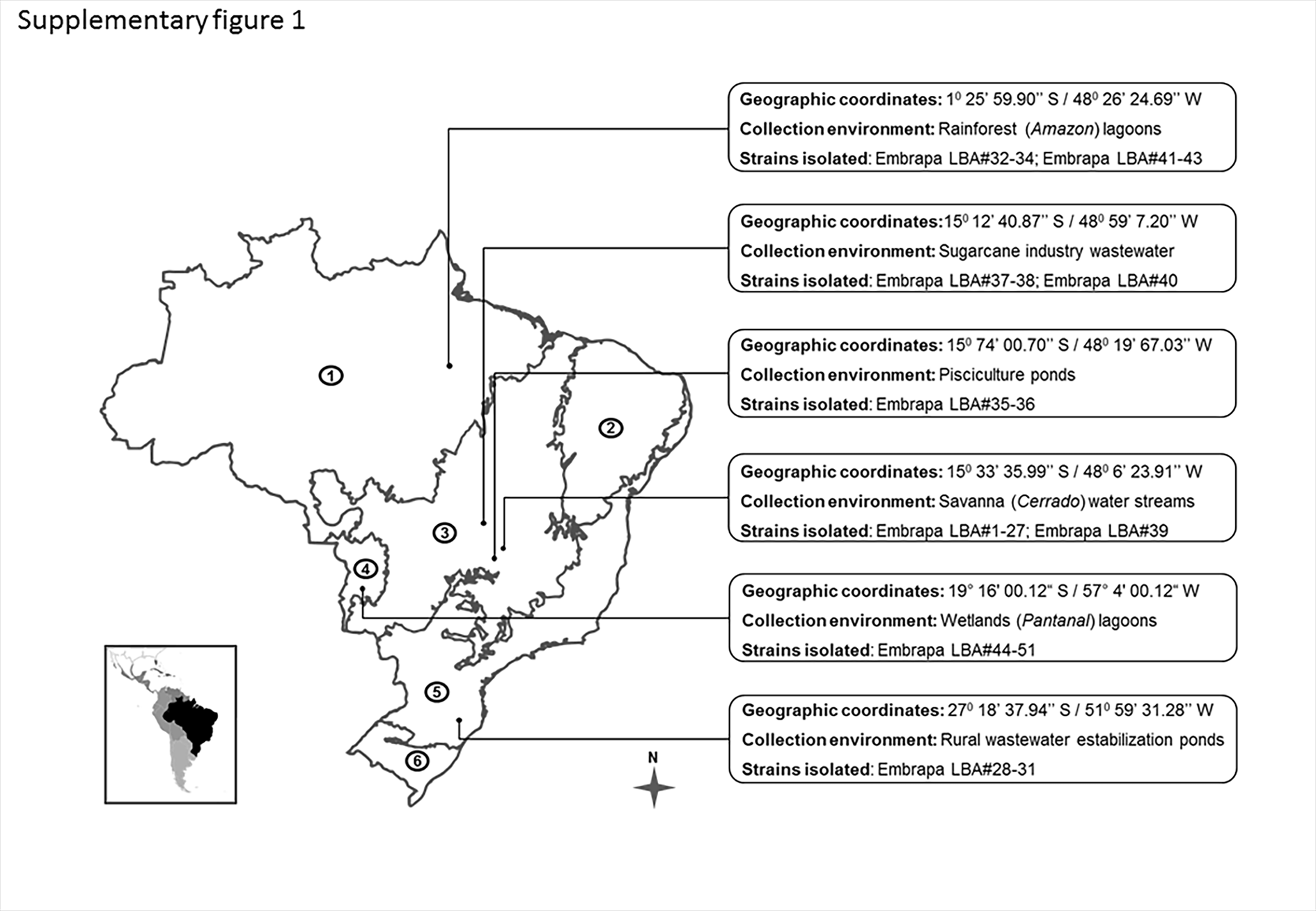

Supplement: S1 Fig — Map of Brazilian biomes, including the Amazon tropical rainforest (1), the Caatinga xeric shrublands (2), the Cerrado tropical Savanna (3), the Pantanal flooded grassland (4), the Mata Atlântica tropical rainforest (5) and the Pampa subtropical grassland (6). The geographic coordinates of the six distinct locations sampled and the respective isolated strains in each site are shown. The strains isolated were deposited in the Collection of Microorganisms and Microalgae Applied to Agroenergy and Biorefineries at Embrapa (Brasília/DF–Brazil). The Brazilian territory is highlighted in black in the map of the neotropical region (inset). (TIF) [file pone.0149284.s001.tif]

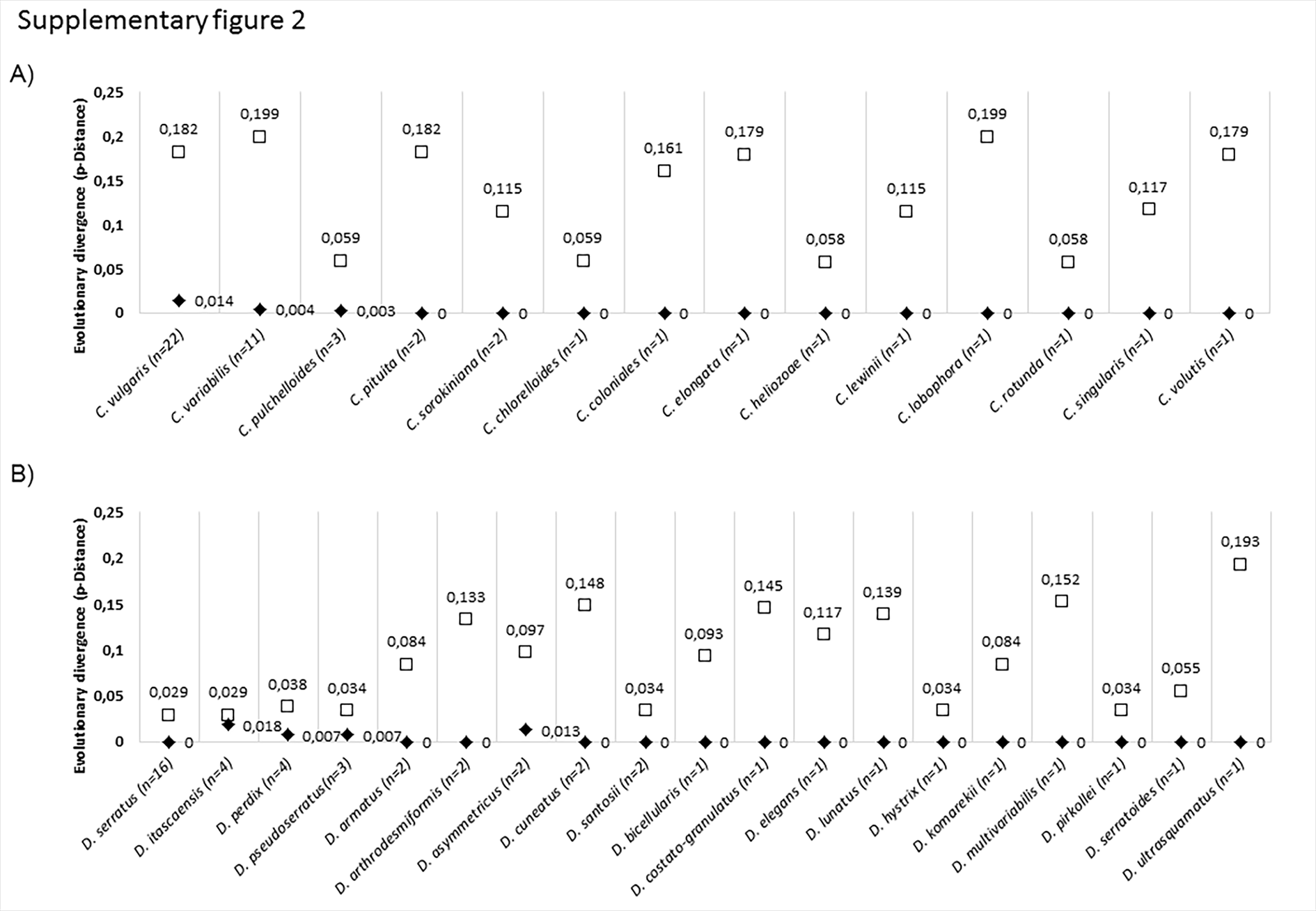

Supplement: S2 Fig — The maximum intraspecific distances (◆) and minimum interspecific distances (□) based on nuITS1 marker between Chlorella (A) and Desmodesmus (B) genera species are shown. The dataset was composed of reference barcode sequences reported for each genera (S1 and S2 Tables). (TIF) [file pone.0149284.s002.tif]

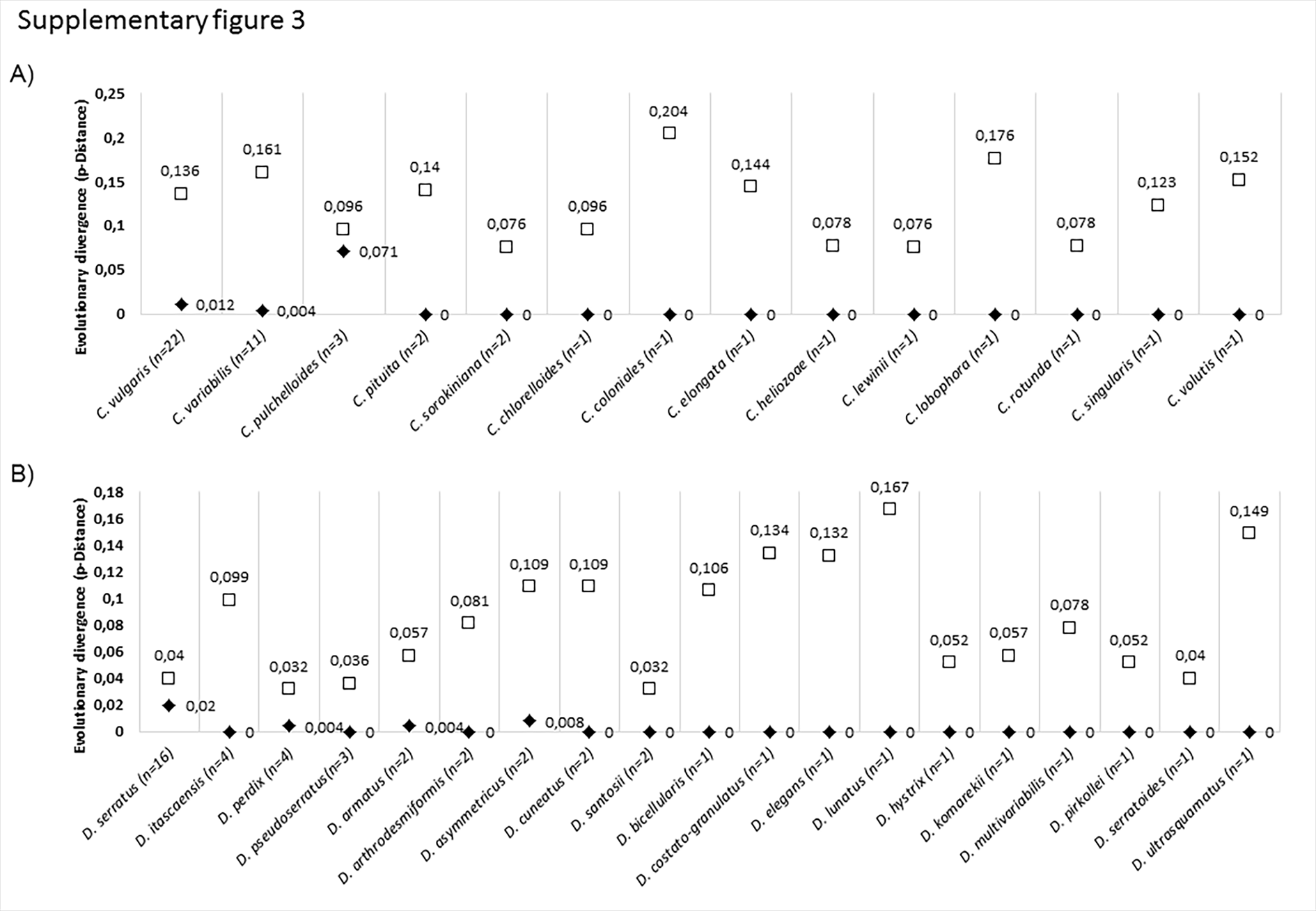

Supplement: S3 Fig — The maximum intraspecific distances (◆) and minimum interspecific distances (□) based on nuITS2 marker between Chlorella (A) and Desmodesmus (B) genera species are shown. The dataset was composed of reference barcode sequences reported for each genera (S1 and S2 Tables). (TIF) [file pone.0149284.s003.tif]

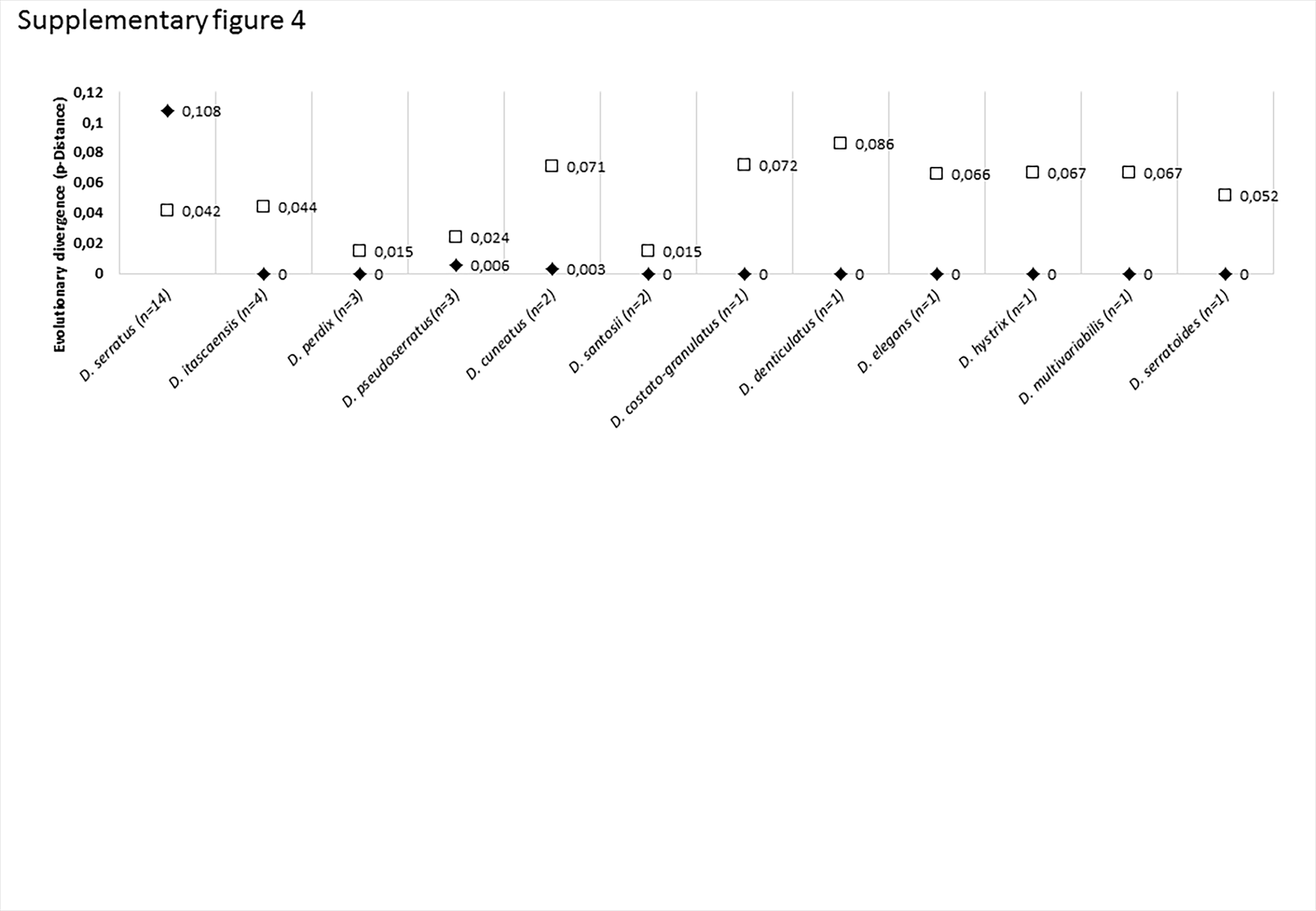

Supplement: S4 Fig — The maximum intraspecific distances (◆) and minimum interspecific distances (□) based on rbcL marker between Desmodesmus genus species are shown. The dataset was composed of reference barcode sequences reported this genus (S3 Table). (TIF) [file pone.0149284.s004.tif]

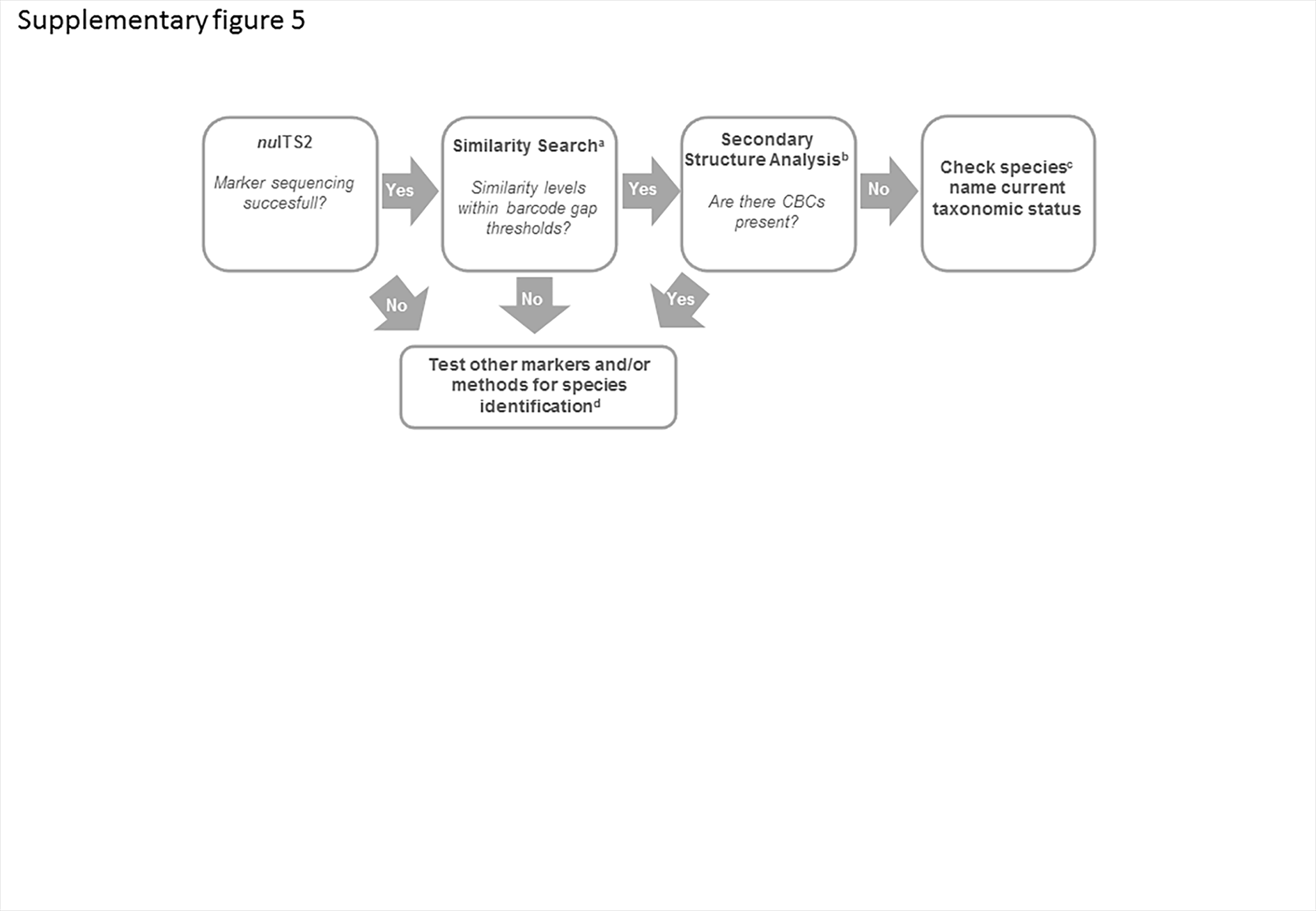

Supplement: S5 Fig — nuITS2 should be primarily sequenced and submitted to similarity searches against GenBank. Similarity values obtained must be compatible with the barcode gap thresholds calculated using reference sequences for the taxon indicated (a). The absence of CBCs between the query nuITS2 sequence and its closest match retrieved from similarity search is necessary to confirm species diagnosis (b). Finally, the current status of the assigned species name must be checked using a reference database (e.g.: AlgaeBase) (c). If nuITS2 is not sufficient for a species diagnosis, other markers/methods should be tried (d). (TIF) [file pone.0149284.s005.tif]
