# Supplementary material for: DNA Barcoding Green Microalgae Isolated from Neotropical Inland Waters
Source: PLoS One. 2016 Feb 22;11(2):e0149284. doi: 10.1371/journal.pone.0149284 (PMC4767179; doi:10.1371/journal.pone.0149284)
Supplement: S1 Table — (DOCX) [file pone.0149284.s006.docx]

| **Species** | **Accession Number** | **Reference** |
| --- | --- | --- |
| *Chlorella chlorelloides* | HQ111432 | [14] |
| *Chlorella coloniales* | FM205862 | [39] |
| *Chlorella elongata* | FM205858 | [39] |
| *Chlorella heliozoae* | FM205850 | [39] |
| *Chlorella lewinii* | FM205861 | [14] |
| *Chlorella lobophora* | FM205833 | [39] |
| *Chlorella pituita* | FM205856, GQ176853 | [39] |
| *Chlorella pulchelloides* | HQ111430, HQ111431, FM205857 | [14, 39] |
| *Chlorella rotunda* | HQ111433 | [14] |
| *Chlorella singularis* | HQ111435 | [14] |
| *Chlorella sorokiniana* | FM205860, FM205859 | [39] |
| *Chlorella variabilis* | AB162913, AB162912, AB206546, AB206550, AB162914, AB162915, AB162916, AB162917, AB219527, AB206549, FM205849 | [39, 59] |
| *Chlorella volutis* | HQ111434 | [14] |
| *Chlorella vulgaris* | AY591508, AY591509, AY591510, AY591511, AY591512, AY591513, AY591500, AY591501, AY591502, AY591503, AY591504, AY591505, AY591506, AY591493, AY591494, AY591495, AY591496, AY591497, AY591498, AY591499, AB162910, FM205854 | [39, 59] |
