# Supplementary material for: DNA Barcoding Green Microalgae Isolated from Neotropical Inland Waters
Source: PLoS One. 2016 Feb 22;11(2):e0149284. doi: 10.1371/journal.pone.0149284 (PMC4767179; doi:10.1371/journal.pone.0149284)
Supplement: S2 Table — (DOCX) [file pone.0149284.s007.docx]

| **Species** | **Accession Number** | **Reference** |
| --- | --- | --- |
| *Desmodesmus armatus var. subalternans* | DQ417520, DQ417547 | [54] |
| *Desmodesmus arthrodesmiformis* | DQ417534, DQ417535 | [54] |
| *Desmodesmus asymmetricus* | DQ417549, DQ417576 | [54] |
| *Desmodesmus cuneatus* | DQ417566, DQ417567 | [54] |
| *Desmodesmus bicellularis* | DQ417558 | [54] |
| *Desmodesmus hystrix* | DQ417551 | [54] |
| *Desmodesmus komarekii* | DQ417562 | [54] |
| *Desmodesmus multivariabilis var. turskensis* | DQ417525 | [54] |
| *Desmodesmus pirkollei* | DQ417557 | [54] |
| *Desmodesmus serratus* | GU192371, GU192372, GU192373, GU192374, GU192375, GU192376, GU192377, GU192378, GU192379, GU192380, GU192381, GU192382, GU192383, DQ417559, DQ417560, DQ417561 | [53] |
| *Desmodesmus itascaensis* | DQ417538, DQ417539, DQ417540, DQ417541 | [53] |
| *Desmodesmus perdix* | GU192385, GU192386, GU192387, DQ417573 | [53] |
| *Desmodesmus pseudoserratus* | GU192389, GU192390, GU192391 | [53] |
| *Desmodesmus santosii* | GU192388, DQ417524 | [53] |
| *Desmodesmus costato-granulatus* | DQ417574 | [53] |
| *Desmodesmus elegans* | DQ417581 | [53] |
| *Desmodesmus lunatus* | GU192393 | [53] |
| *Desmodesmus serratoides* | GU192384 | [53] |
| *Desmodesmus ultrasquamatus* | GU192392 | [53] |
