# Supplementary material for: DNA Barcoding Green Microalgae Isolated from Neotropical Inland Waters
Source: PLoS One. 2016 Feb 22;11(2):e0149284. doi: 10.1371/journal.pone.0149284 (PMC4767179; doi:10.1371/journal.pone.0149284)
Supplement: S3 Table — (DOCX) [file pone.0149284.s008.docx]

| **Species** | **Accession Number** | **Reference** |
| --- | --- | --- |
| *Desmodesmus cuneatus* | GU192435, GU192436 | [54] |
| *Desmodesmus costato-granulatus* | GU192427 | [54] |
| *Desmodesmus denticulatus var. linearis* | GU192429 | [54] |
| *Desmodesmus hystrix* | GU192433 | [54] |
| *Desmodesmus multivariabilis var. turskensis* | GU192431 | [54] |
| *Desmodesmus serratoides* | GU192413 | [54] |
| *Desmodesmus serratus* | GU192399, GU192400, GU192401, GU192402, GU192403, GU192404, GU192405, GU192406, GU192407, GU192408, GU192409, GU192410, GU192411, GU192412 | [53] |
| *Desmodesmus itascaensis* | GU192422, GU192423, GU192424, GU192425 | [53] |
| *Desmodesmus perdix* | GU192414, GU192415, GU192416 | [53] |
| *Desmodesmus pseudoserratus* | GU192419, GU192420, GU192421 | [53] |
| *Desmodesmus santosii* | GU192417, GU192418 | [53] |
| *Desmodesmus elegans* | GU192426 | [53] |
